# Histopathological characteristics of pulmonary emphysema in experimental model

**DOI:** 10.1590/S1679-45082014AI2681

**Published:** 2014

**Authors:** Antonio Di Petta

**Affiliations:** 1 Universidade de São Paulo, São Paulo, SP, Brazil.

Historically pulmonary emphysema was described in 1834 by Laennec on the basis of observations made on the cut surface of postmortem human lungs being the lesion attributed to the atrophy of lung tissue from pulmonary hyperinflation.^(1)^ Hence, emphysema was redefined as a “abnormal and permanent dilation of distal air spaces of terminal bronchiole”.^(2)^ In addition, evidences of destruction of alveolar wall and fibrosis must not be ignored in this disease pathogenesis.^(3)^

These anatomopathological changes result in loss of respiratory surface and blood irrigation, decrease of elastic recognition and pulmonary hyperexpansion, and it could also affect part of acinus or its structure.^(4)^

Pulmonary emphysema is caused by enzymatic imbalance between proteases and anti-proteases that results in destruction of the alveolar wall due to proteolytic enzymes action, which affects the extracellular matrix (ECM)^(5)^ and its component integrity especially the elastic fibres.^(6)^

Experimental model of pulmonary emphysema is based on nebulization or instillation of proteolytic enzyme, such as panain *(Carica papaya)*,^(7)^ porcine pancreatic elastase,^(4)^ and human neutrophil elastase.^(8)^ This proteolytic process, associated with uniform destruction of ECM of pulmonary acinus, ends up in morphohistological and physiological changes in lungs that resemble those changes find in emphysema in humans.^(9,10)^

**Figure 1:**
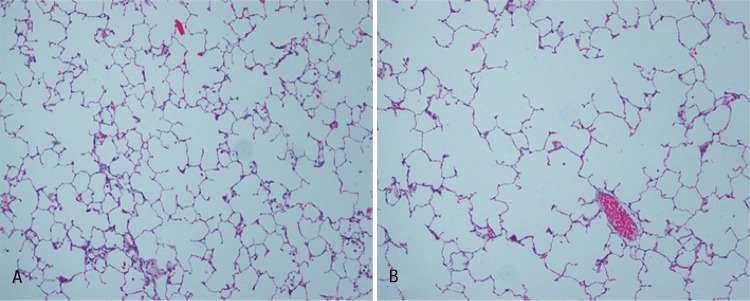
Photomicrographs of lung parenchyma (hematoxylin-eosin) x 100 increased. (A) Naïve lung and (B) emphysematous lung showing hyperdistension of alveolar ducts associated with the rupture of alveolar septa

**Figure 2:**
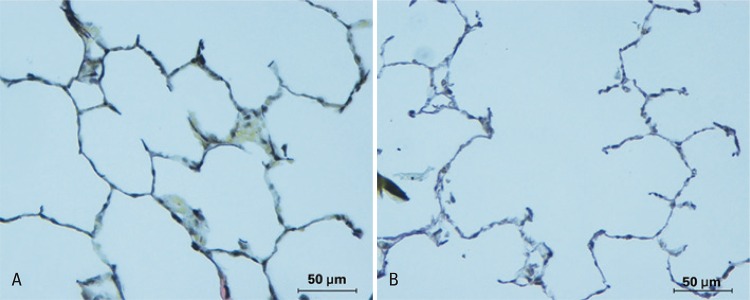
Photomicrographs of lung parenchyma (Verhoeff), x 400 increased. Lung naïve showing integrity of elastic component of alveolar wall, opposing to areas revealed throughout septa associated with thickening of elastic fibres in alveolar wall and decreasing of proportion of elastic fibres in emphysematous lung (B)

Dilatation of distal air spaces of terminal bronchiole (Figure 1) and reduction of area occupied by elastic fibres (Figure 2) evidenced histologically the pulmonary emphysema in experimental models that use porcine pancreatic elastase.
