# Supplementary material for: Histopathological characteristics of pulmonary emphysema in experimental model
Source: Einstein (Sao Paulo). 2014 Jul-Sep;12(3):382–3. doi: 10.1590/S1679-45082014AI2681 (PMC4872956; doi:10.1590/S1679-45082014AI2681)
Supplement: Translation

# Translation: Características histopatológicas do enfisema pulmonar em modelo experimental

**Authors:** Antonio Di Petta

**Affiliations:** 1 Universidade de São Paulo, São Paulo, SP, Brasil.

Historicamente, Laennec (1834) descreveu o enfisema pulmonar a partir de observações em cortes necroscópicos superficiais de pulmões humanos, atribuindo a lesão à atrofia do tecido pulmonar resultante da hiperinsuflação pulmonar.^(1)^ O enfisema foi, então, redefinido como uma “dilatação anormal e permanente dos espaços aéreos distais do bronquíolo terminal”.^(2)^ Além do mais, a evidência da destruição da parede alveolar e de fibrose não deve ser ignorada na patogenia da doença.^(3)^

Essas alterações anatomopatológicas resultam na perda da superfície respiratória e de irrigação sanguínea, na diminuição do recolhimento elástico e na hiperexpansão pulmonar, podendo atingir parte do ácino ou toda sua estrutura.^(4)^

O enfisema pulmonar é obviamente uma doença causada pelo desequilíbrio enzimático existente entre proteases e antiproteases, resultando na destruição da parede alveolar ocasionada pela ação de enzimas proteolíticas, que degradam a matriz extracelular (MEC)^(5)^ e afetam a integridade de seus componentes, particularmente as fibras elástica.^(6)^

Modelos experimentais de enfisema pulmonar baseiam-se na nebulização ou instilação de enzimas proteolíticas, como papaína (*Carica papaya*),^(7)^ elastase pancreática de porco,^(4)^ e elastase neutrofílica humana.^(8)^ Esse processo proteolítico, associado à destruição uniforme da MEC do ácino pulmonar, resulta em alterações morfo-histológicas e fisiológicas dos pulmões equivalentes às alterações encontradas no enfisema em seres humanos.^(9,10)^

A dilatação dos espaços aéreos distais do bronquíolo terminal (Figura 1) e a redução da área ocupada pelas fibras elásticas (Figura 2) evidenciam histologicamente o enfisema pulmonar em modelos experimentais instilados por elastase pancreática de porco.

**Figura 1:** Fotomicrografias do parênquima pulmonar (hematoxilina-eosina), aumento x100. (A) Pulmão *naive* e (B) pulmão enfisematoso, demonstrando hiperdistensão dos ductos alveolares com ruptura dos septos alveolares

**Figura 2:** Fotomicrografias do parênquima pulmonar (*Verhoeff*), aumento de 400x. (A) Pulmão *naive* demonstrando integridade dos componentes elásticos da parede alveolar, contrastando com áreas desnudas ao longo dos septos associadas ao adensamento de fibras elásticas na parede alveolar e diminuição da concentração de fibras elásticas no pulmão enfisematoso (B)
